# Sliding limbus-conjunctival flaps for minimizing the pterygium recurrence: a prospective tertiary care institute study

**DOI:** 10.22336/rjo.2023.41

**Published:** 2023

**Authors:** Manpreet Kaur, Manpreet Singh, Shakeen Singh

**Affiliations:** * Department of Ophthalmology, Sri Guru Ram Das Institute of Medical Sciences and Research, Amritsar, India; ** Department of Ophthalmology, Advanced Eye Centre, Post Graduate Institute of Medical Education and Research, Chandigarh, India

**Keywords:** pterygium, pterygium recurrence, pterygium surgery, sliding conjunctival flaps

## Abstract

**Objective:** To describe the surgical technique and long-term outcomes of sliding limbus-conjunctival flaps to treat primary pterygium.

**Methods:** Our single-center, single-surgeon-based, prospective study (part of the postgraduate thesis) included primary pterygium patients. We included the larger pterygium in bilateral cases for our research. All patients underwent pterygium excision and reconstruction by sliding limbus-conjunctival flaps technique to minimize the recurrence rates. Similar surgical steps and drugs were used for all the enrolled patients. Two ophthalmologists (SS and MK) evaluated all cases for blinding purposes. A minimum follow-up of 12 months was ensured in all cases.

**Results:** Fifty eyes of 50 patients underwent the sliding limbus-conjunctival flaps surgery at a mean age of 50.40 ± 15.05 years. There were 27 (54%) males and 23 (46%) females with nasal pterygium (100%), having an average horizontal size of 2.96 mm. A “with the rule” astigmatism was seen in 44 (88%) eyes with a horizontal keratometry value of 42.00 ± 2.83, which increased significantly to 42.23 ± 2.02 (p>0.05) after surgery. The visual acuity was improved by 1 Snellen’s line in 21 cases, 2 lines in 4 cases, and by 3 lines in 1 case. No change in visual acuity was seen in 24 cases. The early minor postoperative complications were self-resolving. At a mean follow-up of 14.5 months, the recurrence was found in 2 patients (4%), one in the 3rd and the other in the 9th month.

**Conclusion:** The sliding limbus-conjunctival flaps technique is a simple, safe, and efficient procedure for primary pterygium cases. Pterygium surgery positively affects the visual acuity and keratometry values in most patients, making it a cosmetic and functional ophthalmic surgical procedure.

## Introduction

Pterygium is a fibro-vascular growth of actinically damaged conjunctiva encroaching upon the cornea, across the limbus within the interpalpebral fissure, commonly on the nasal side [**1**]. A recent Indian study found positive associations of pterygium with the coastal location, illiteracy, and increasing lifetime sun exposure. They found a negative association of pterygium with BMI ≥ 25 kg/m2 [**1**]. Pterygium presents as an oculofacial cosmetic blemish and, in large size, may lead to optical anomalies like astigmatism due to the distortion of corneal topography [**2**]. In advanced cases, it may also obscure the optical center of the cornea, necessitating its urgent treatment.

Surgery is considered the gold standard for the treatment of pterygium. However, the main concern and challenge for the operating surgeon is preventing pterygium recurrence. To avoid the recurrence of pterygium, the adjunctive use of mitomycin-C (MMC), 5-fluorouracil (5-FU), cyclosporine, beta irradiation, and argon laser application have been used with considerable success [**3**]. However, modifications in surgical techniques like conjunctival autograft, conjunctival limbal autograft, and amniotic membrane transplantation significantly reduce the recurrence rate [**4**]. Extended tenon resection techniques have also been described to minimize the fibrotic recurrence of the pterygium [**5**]. These techniques aim to provide “healthy” conjunctival tissue, debulk fibrotic tissue, and present a limbus stem cell “barrier” in the pterygium zone [**1**-**5**].

Fixing conjunctival flaps has been described as another factor in preventing recurrence by using electrocautery-based closure or autologous serum [**6**,**7**]. Avoiding the use of an essential autologous tissue (conjunctiva) and lengthy surgical procedures, along with providing a cost-effective alternative, drove us to conduct our study. We aimed to provide a simple, easy, and efficient surgical technique using the neighboring conjunctiva-limbus flaps to minimize pterygium recurrence.

## Methods

This prospective, non-comparative, single-center, single-surgeon (SS) clinical study included patients with primary pterygium. The study period was from January 2013 to December 2014, i.e. 2 years. Our study was approved by the Institute Ethics Committee and adhered to the tenets of the Declaration of Helsinki. Written informed consent was obtained from all the participants after describing the technique to them. All consecutively enrolled patients with primary pterygium located in the nasal quadrant and the larger pterygium were registered for our study in bilateral cases. The exclusion criteria were recurrent pterygium, pseudo-pterygium, marginal healed corneal ulcer with vascularization, symblepharon, post-chemical injury, and ophthalmic trauma or surgery.

The comprehensive ophthalmic examination included best-corrected visual acuity (BCVA), Goldmann applanation tonometry, slit-lamp examination including the type and size of pterygium, keratometry, and fundus examination, which were performed for all the participants. The size of the pterygium was measured in millimeters (mm) using a Vernier caliper. The horizontal extent of pterygium, i.e., encroachment of cornea, was noted and graded as < 2mm = grade 1, ≥ 2mm to 4mm = grade 2, and > 4mm = grade 3.

*Surgical technique*

**Fig. 1:**
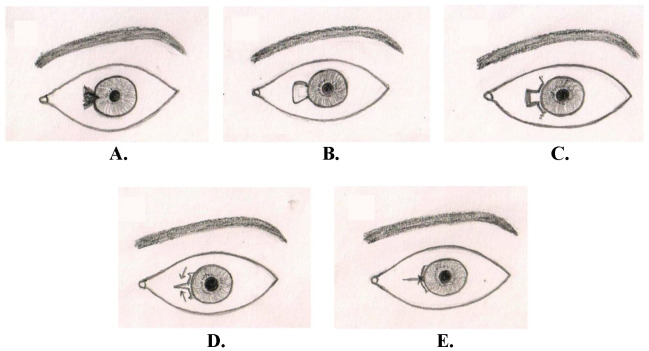
Cartoon of the pterygium surgical excision and sliding limbus-conjunctival graft. **A.** Left eye with nasal pterygium; **B.** After complete removal, measure the bare sclera at the limbus; **C.** Fashioning the superior and inferior conjunctival-limbus flaps and radial cuts at distal ends to mobilize the flaps; **D.** Mobilization of superior and inferior flaps to cover the bare area; **E.** Fixation of the flaps in the center using a single suture and the patient’s plasma

All procedures were performed by a single surgeon (SS) and assisted by the same person (MK). After proper surgical cleaning and draping, a universal eyelid speculum was applied. Topical proparacaine 0.5% eye drops were instilled, followed by the irrigation of conjunctival cul-de-sac with 5% povidone-iodine solution and normal saline. Local subconjunctival anesthesia (1 ml) was injected with 26 gauze needles under the conjunctival layer of the pterygium body to “hydro dissect” it from underlying fibro-vascular tissue. The ballooned conjunctiva was cut vertically over the limbus and dissected free from the underlying fibrous tissue. The exposed fibro-vascular layer of pterygium was removed using scissors and a crescent blade from the scleral bed. The head of the pterygium was avulsed using fine-toothed forceps assisted with a crescent blade. The involved conjunctival and limbal tissue was also removed using scissors, leaving behind a bare sclera (**Fig. 1A, B**).

The length of the excised limbus was measured with Castroveijo’s calipers (x). At that moment, two conjunctival flaps, including normal limbus, were fashioned superiorly and inferiorly with tangential lengths of half the x i.e. x/2 (**Fig. 1C**). Hence, the length of each limbus flap was divided equally to cover the excised limbus zone completely. Small radial relaxing incisions were given at the distal ends of limbus-conjunctival flaps to facilitate their sliding (**Fig. 1C, D**). After the mobilization of the flaps, a single suture at the medial limbus was applied to both flaps and fixed to the scleral bed (**Fig. 1E**). The remaining gap between the flaps was filled by pulling them together and fixed with the patient’s plasma from the local bleed. Antibiotic ointment (ciprofloxacin 0.3%) was applied over the conjunctiva to ensure smoother closure and opening of eyelids and minimize mechanical stretch.

**Fig. 2:**
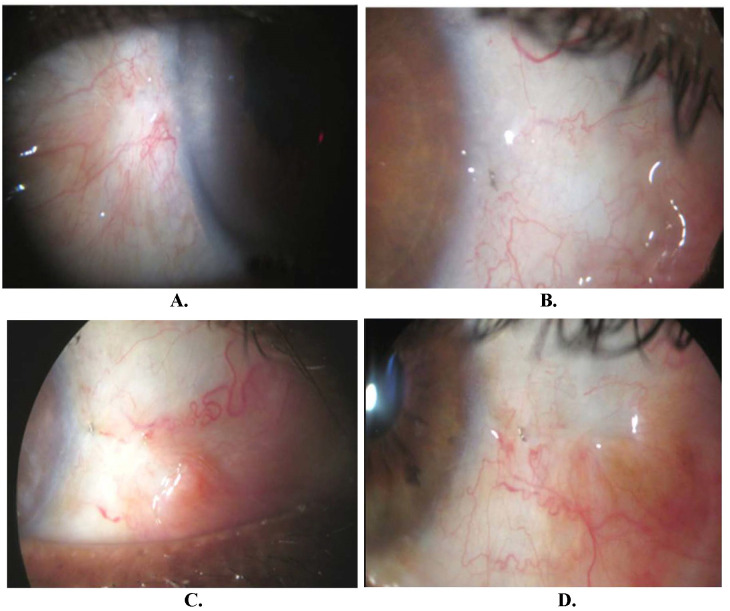
Long-term follow-up of cases. **A.** Left eye with healed pterygium area having vascularization; **B.** Right eye showing quiet zone of healing and a single steel suture in place; **C.** Well-healed pterygium site of the right eye with no vascularization; **D.** Nicely healed area after removal of right eye nasal pterygium with single steel suture in place

Postoperatively, eyedrops moxifloxacin 0.5% and dexamethasone 0.1% combination (4 times/day), eyedrops carboxymethylcellulose 1% (6 times/day), and moxifloxacin eye ointment 0.3% during the night was prescribed to all patients. The patients were followed up on days 1, 7, 14, and 30, at 2 months, 3 months, 6 months, and 12 months. The visual acuity, intraocular pressure, and keratometry values were recorded at each visit. Complete success was defined as total removal of pterygium + no vascularization at the flap site + no patient discomfort. Partial success was defined as total removal of pterygium + vascularization of > 50% flap site + no/mild patient discomfort. Treatment failure was defined as recurrence of pterygium + vascularization > 50% flap site + patient discomfort (**Fig. 2**). 

The Statistical Package for the Social Sciences program (IBM SPSS version 21.0 for Windows, Chicago, IL, USA) was used for statistical analysis. Mean ± SD presented descriptive statistics. The student t-test was used for quantitative variables and the chi-square test for qualitative variables. The “p-value” of < 0.05 was considered significant.

## Results

We enrolled 50 patients having primary pterygium in 50 eyes. There were 27 (54%) males and 23 (46%) females, with 33 (66%) patients having unilateral and 17 (34%) having bilateral pterygium. At presentation, the mean age was 50.40 ± 15.05 years. All patients had nasal pterygium and equal right and left eye distribution. The average horizontal size of pterygium was 2.96 ± 1.08 mm, with < 2 mm in 6 eyes (12%) and ≥ 2-4 mm in 44 eyes (88%).

The pre-operative visual acuity of ≥ 6/12 was recorded in 42 eyes (84%). Forty-four eyes (88%) had “with-the-rule” astigmatism, 4 eyes (8%) had “against-the-rule” astigmatism, while 2 eyes (4%) showed no astigmatic error. In refractive error, hypermetropia was noted in 32 eyes (64%), 14 eyes (28%) were myopic, and emmetropia was present in 4 (8%) eyes. 

All patients had intraocular pressure in the normal range i.e. < 20 mm Hg and none had any history of glaucoma in their families. All patients underwent the sliding limbus-conjunctival flaps for the nasal pterygium in the prescribed format. In 5 eyes, additional use of a crescent blade was needed to remove the head of the pterygium from the corneal surface, while it was required for most cases while working in the scleral region. Postoperatively, the visual acuity improved by 1 line in 21 cases, by 2 lines in 4 cases, and by 3 lines in 1 case. No change in visual acuity was seen in 24 cases. Keratometry values (K1 readings) increased from 42.00 ± 2.83 to 42.23 ± 2.02 (p>0.05) after surgery, while K2 reading changed from 43.86 ± 1.89 to 43.88 ± 1.88 (p˃0.05). There was an average reduction of 0.79 ± 1.25D in the magnitude of spherical and 0.76 ± 1.15D in the magnitude of the cylindrical element of refraction after surgery.

No major intraoperative complications were noted. The early postoperative complications included pain and discomfort in 11 patients, irritation and foreign body sensation in 34 patients, and lacrimation in 41 patients, and all these issues were self-resolving. At 3 weeks follow-up, the flap retraction was seen in one patient who required additional sutures. 

At the mean follow-up of 14.5 months, complete success was achieved in 38 eyes, while partial success was noted in 10 eyes. Treatment failure in the form of pterygium recurrence was noted in 2 eyes (4%). In one patient, recurrence was noted at 3rd month and in another at 9th month. Both had bilateral pterygium, and their 1 eye was included in our study. On further investigation, both had a history of excessive sun exposure (> 8 hours/day) as they worked in the field as daily wagers. 

## Discussion

Pterygium surgery focuses on minimizing the recurrence rate and avoiding subsequent secondary surgeries or procedures. The modifiable risk factors include environmental factors- prolonged exposure to sun rays and chemical compounds [**1**,**3**,**8**]. Generally, our limbal stem cell in health provides a natural barrier for the conjunctival epithelium to grow over the corneal epithelium. Hence, we focused on moving or sliding the surrounding limbal stem cells via the limbus-conjunctival graft to minimize pterygium recurrence [**6**-**11**].

To cover the excised pterygium area and minimize the collateral damage to the surrounding conjunctival mucosa, people have used amniotic membranes and cultivated membranes to cover the area with variable success [**4**,**9**]. In these cases, the limbal stem cells are not transported to the damaged area, making it prone to recurrence [**4**,**8**].

We followed a simple, effective, easy, and time-saving technique of limbus-conjunctival flaps and modified it slightly compared to described surgical procedures [**9**-**12**]. We used a single suture at the medial limbus to fix these flaps to the underlying sclera to avoid flap retraction in the early postoperative period. In a similar study by Lee et al. (2021), > 1 suture were used to fix the superior and inferior conjunctival flaps, which may lead to more inflammation and possible sequels [**10**]. We made radial incisions at the distal ends of both the superior and inferior conjunctival flaps for easy mobilization and to fix them without undue tension. 

Kurna SA et al. compared limbal sliding flap transplantation (28 eyes) with 2 other surgical treatment options, i.e., primary closing (25 eyes), and amniotic membrane grafting (22 eyes). Postoperatively, they found that corneal astigmatism decreased and keratometry values increased significantly in all the groups, with no statistically significant difference. They concluded that the limbal-conjunctival sliding flap transplantation is a safe, efficient, and effective method to manage primary pterygium, minimizing the recurrence risk (7.1%). Our study found a recurrence rate of 4% (2 patients) seen over nine months of follow-up compared to the above-mentioned studies [**9**].

McCoombes JA (1994) described a similar method of sliding only the superior limbal conjunctival flap for treating the primary pterygium in 258 eyes with a 3.2% recurrence rate, with early postoperative flap retraction being a common reason for recurrence [**11**]. Other researchers have used similar techniques to mobilize the limbal conjunctival flaps near the removed pterygium [**8**-**13**].

The P.E.R.F.E.C.T (pterygium extended removal followed by extended conjunctival transplant) technique of pterygium excision described by Hirst et al. has shown a < 0.1% recurrence rate [**5**]. They focused on and described that careful conjunctival dissection without involving tenons in the graft could help decrease the recurrence rate and flap retraction. We followed similar principles during the surgical excision of the pterygium using hydrodissection to create a dissection plane, to minimize the recurrence in our patients.

In complications, McCoombes JA et al. noted flap retraction in the immediate postoperative period in 11 (5%) eyes, the majority belonging to the recurrence group. They reported the early postoperative flap retraction as the likely factor for recurrences at a mean interval time of 4.3 months. In a recently conducted analysis of complication profile after pterygium excision in primary and recurrence pterygium, Kodavoor et al. mentioned sub-conjunctival hemorrhage in 912 eyes (38.7%), edema of the graft in 522 cases (22.15%), and graft retraction in 692 cases (29.37%) as the major complications. Graft loss (0.93%), graft sliding (0.38%) and granuloma at the host site (0.16%) were described as the less common complications. We believe that the use of adjacent superior and inferior conjunctival-limbus flaps may improve wound healing, reduce complications, and preserve the superior conjunctiva for future glaucoma surgeries if needed. **Table 1** compares various studies focused on the time of recurrence of pterygium after surgery.

**Table 1:** Comparison of surgical technique, recurrences, and follow-up by various authors

Sr. No.	Author (year)	Eyes	Surgical technique	Recurrence	The average period of recurrence (months)	Follow-up (months)
1	McCoombes et al. (1994)	258	Superior sliding conjunctival flap	7 (3.2%)	4.3	12
2	Tomas et al. (1990)	20	Sliding limbal conjunctiva flap	1 (5%)	-	12
3	Ali et al. (2001)	126	Sliding conjunctival flap	6 (4.7%)	2.1	24
4	Kurna et al. (2013)	28	Limbal-conjunctival sliding flap - 28, Amniotic membrane grafting - 22, Primary closure - 25	2 (7.1%), 6 (27.3%), 14 (56%)	4th month, 4.4 month, 4.4 month	15, 20, 28
5	Present study (2023)	50	Sliding limbal conjunctival flap	2 (4%)	1 by 3rd month and 1 by 6th month	14.5

The pterygium can affect the central cornea if it covers > 45% of the corneal radius or it reaches within 3.2 mm of the visual axis [**14**]. This leads to “with the rule” astigmatism, which is hemi-meridional on the side of the pterygium in most cases [**14**]. The optics of astigmatism can be explained by the pooling of tear film at the leading edge of the pterygium or due to traction by the fibrovascular tissue of the pterygium, resulting in the flattening of the cornea in the horizontal meridian [**15**,**16**]. In our study, after pterygium surgery, the keratometry value of the horizontal meridian increased from 42.00 ± 2.83 to 42.23 ± 2.02 (p> 0.05) in most of the cases resulting in a decrease in the magnitude of “with the rule” astigmatism or “no astigmatism”, and keratometry of vertical meridian increased in 5 and decreased in 3 cases, while 7 cases showed no change. The increase in vertical curvature can be secondary to the pressure exerted by the eyelids over the cornea due to ocular surface inflammation related to increasing blinking with force. We believe that the improvement in visual acuity in most cases was attributed to the reduction of astigmatism.

## Conclusion

In conclusion, the sliding limbus-conjunctival flaps (superior and inferior) are a simple, easy, and efficient technique to minimize the recurrence rate after primary pterygium surgery. We found a single limbus suture sufficient to retain the flaps’ strength in the middle. Pterygium surgery leads to improvement in visual outcomes secondary to corneal astigmatism.

**Conflict of Interest statement**

The authors declare no conflict of interest.

**Informed Consent and Human and Animal Rights statement**

Informed consent has been obtained from all individuals included in this study.

**Authorization for the use of human subjects**

Ethical approval: The research related to human use complies with all the relevant national regulations and institutional policies, is by the tenets of the Helsinki Declaration, and has been approved by the Ethics Committee of Sri Guru Ram Das Institute of Medical Sciences and Research, Amritsar, India.

**Acknowledgments**

To our patients, who believed in us to deliver the best for their eye conditions.

**Sources of Funding**

None.

**Disclosures**

None.

**Meeting presentation**

None.
